# Distinguishing thymic cysts from low-risk thymomas via [^18^F]FDG PET/CT

**DOI:** 10.1186/s13550-024-01108-3

**Published:** 2024-05-03

**Authors:** Sunju Choi, Yong-il Kim, Sangwon Han, Jae Kwang Yun, Geun Dong Lee, Sehoon Choi, Hyeong Ryul Kim, Yong-Hee Kim, Dong Kwan Kim, Seung-Il Park, Jin-Sook Ryu

**Affiliations:** 1 Department of Nuclear Medicine, Asan Medical Center, University of Ulsan College of Medicine, Seoul, Republic of Korea; 2 Department of Nuclear Medicine, Kyung Hee University Hospital, School of Medicine, Kyung Hee University, Seoul, Republic of Korea; 3 Department of Thoracic and Cardiovascular Surgery, Asan Medical Center, University of Ulsan College of Medicine, Seoul, Republic of Korea

**Keywords:** Thymus neoplasms, Fluorodexoyglucose F18, Positron Emission Tomography Computed Tomography, Sensitivity and specificity

## Abstract

**Background:**

Thymic cysts are a rare benign disease that needs to be distinguished from low-risk thymoma. [^18^F]fluorodeoxyglucose (FDG) positron emission tomography (PET)/computed tomography (CT) is a non-invasive imaging technique used in the differential diagnosis of thymic epithelial tumours, but its usefulness for thymic cysts remains unclear. Our study evaluated the utility of visual findings and quantitative parameters of [^18^F]FDG PET/CT for differentiating between thymic cysts and low-risk thymomas.

**Methods:**

Patients who underwent preoperative [^18^F]FDG PET/CT followed by thymectomy for a thymic mass were retrospectively analyzed. The visual [^18^F]FDG PET/CT findings evaluated were PET visual grade, PET central metabolic defect, and CT shape. The quantitative [^18^F]FDG PET/CT parameters evaluated were PET maximum standardized uptake value (SUVmax), CT diameter (cm), and CT attenuation in Hounsfield units (HU). Findings and parameters for differentiating thymic cysts from low-risk thymomas were assessed using Pearson’s chi-square test, the Mann-Whitney U-test, and receiver operating characteristics (ROC) curve analysis.

**Results:**

Seventy patients (18 thymic cysts and 52 low-risk thymomas) were finally included. Visual findings of PET visual grade (*P* < 0.001) and PET central metabolic defect (*P* < 0.001) showed significant differences between thymic cysts and low-risk thymomas, but CT shape did not. Among the quantitative parameters, PET SUVmax (*P* < 0.001), CT diameter (*P* < 0.001), and CT HU (*P* = 0.004) showed significant differences. In ROC analysis, PET SUVmax demonstrated the highest area under the curve (AUC) of 0.996 (*P* < 0.001), with a cut-off of equal to or less than 2.1 having a sensitivity of 100.0% and specificity of 94.2%. The AUC of PET SUVmax was significantly larger than that of CT diameter (*P* = 0.009) and CT HU (*P* = 0.004).

**Conclusions:**

Among the [^18^F]FDG PET/CT parameters examined, low FDG uptake (SUVmax ≤ 2.1, equal to or less than the mediastinum) is a strong diagnostic marker for a thymic cyst. PET visual grade and central metabolic defect are easily accessible findings.

**Supplementary Information:**

The online version contains supplementary material available at 10.1186/s13550-024-01108-3.

## Background

With the wide use of low-dose chest computed tomography (LDCT) screening for lung cancer in patients at high risk, the detection rate for asymptomatic incidental lesions in the anterior mediastinum is increasing [1]. The majority of such lesions originate from the thymus, and their long diameter is usually less than 3 cm [1, 2]. It is both challenging and crucial for the physician to discriminate thymic cyst from small thymic epithelial tumour (TET) on LDCT. TETs are the most common primary neoplasm in the anterior mediastinum in adults [3], and the World Health Organization (WHO) classification categorizes them into five histologic subtypes (thymoma A1, AB, B1, B2, and B3) on the basis of their morphology and degree of atypia, reflecting their invasive nature [4]. Alternative simplified histologic classifications have been suggested, such as categorizing TETs into low-risk thymoma (thymoma A, AB, and B1) and high-risk thymoma (thymoma B2 and B3) [5, 6]. Obviously, the primary concern when dealing with a thymic mass is to ascertain any malignant features, such as invasion into adjacent vascular structures and the presence of suspicious metastatic lesions in the pleura or lung. High-risk thymomas may exhibit distinctive imaging features, such as lobulated or irregular contours and tumour calcifications, unlike low-risk thymomas. Conversely, low-risk thymomas usually appear well-defined, with smooth contours and homogeneous attenuation on CT scans [7–9].

Thymic cysts with CT features showing a unilocular, non-calcified, thin-walled appearance with simple fluid density, especially when asymptomatic, are typically considered benign and can be managed with observation [10–16]. Conversely, cysts lacking these benign radiographic characteristics should be considered for surgical resection. Furthermore, thymic cysts can be identified by a CT attenuation similar to water (≤ 20 Hounsfield units, HU) [11], with such water-similar attenuation being an important imaging characteristic for differentiating thymic cyst from TET on CT scans [7, 11]. However, recent studies revealed that approximately three-fourths of thymic cysts exhibit hyper-attenuation compared to water, potentially leading to misdiagnoses of low-risk thymomas. Clinical misdiagnosis of thymic cyst as low-risk thymoma can lead to a high rate of nontherapeutic thymectomy, thereby subjecting many patients to unnecessary invasive surgery [17, 18].

[^18^F]fluorodeoxyglucose (FDG) positron emission tomography (PET)/computed tomography (CT), which reflects intracellular glycolytic activity, has been evaluated for the differentiation of TETs [19]. However, few studies have investigated the discrimination of thymic cysts from TETs according to the qualitative and quantitative assessment of [^18^F]FDG PET/CT. The aim of this study was therefore to investigate whether [^18^F]FDG PET/CT could help distinguish thymic cyst from low-risk thymoma at the anterior mediastinum.

## Methods

### Patients

**Fig. 1:**
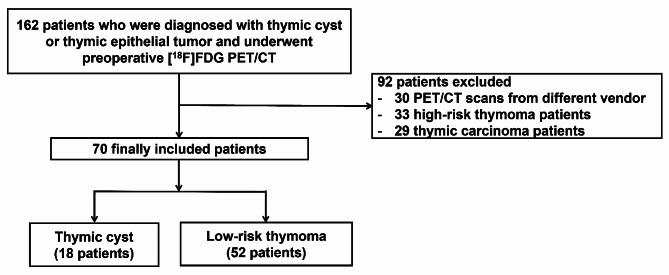
Patient inclusion flow chart

We retrospectively reviewed 162 consecutive patients who underwent preoperative [^18^F]FDG PET/CT as a routine exam and surgical thymectomy for a thymic mass at Asan Medical Center, between January 2014 and December 2021. PET/CT scan from different vendor (30 patients), and cases of high-risk thymoma (33 patients) and thymic carcinoma (29 patients), were excluded. Finally, a total of 70 patients who fulfilled the above criteria were included in the current study (Fig. 1). The study design was approved by the Institutional Review Board of Asan Medical Center (no. 2022 − 1189). Informed consent was waived due to the retrospective nature of the study.

### [^18^F]FDG PET/CT image acquisition

All patients fasted for at least 6 h prior to the PET/CT acquisition, ensuring a venous blood glucose level below 150 mg/dl. Patients received an intravenous injection of 5.18 MBq/kg (range, 114.3–488.4 MBq) of [^18^F]FDG and rested for 60 min before PET/CT scanning. PET imaging was acquired with one of the following scanners, each employing the same resolution and reconstruction parameters: Discovery 690, 710, and 690 Elite (GE Healthcare). During regular quality control, these three PET/CT scanners showed almost similar image quality and SUV values. The Biograph TruePoint 40 scanner—another vendor PET/CT scanner—was excluded from our analysis. This is due to its lower spatial resolution and distinct acquisition and reconstruction parameters, specifically the non-time-of-flight and non-point-spread function.

CT images were initially acquired from the skull base to the upper thigh using the following parameters: 120 kVp, automatic mA, 40 mm collimation, and 3.75 mm slice thickness. PET images of the same area were obtained, allocating 2 min per bed position for 6–7 beds in three-dimensional mode. Data were reconstructed using a 192 × 192 matrix with a voxel size of 2.6 × 2.6 × 3.75 mm, employing an ordered-subset expectation maximization algorithm (18 subsets, four iterations) along with 4.0 mm full-width-at-half-maximum Gaussian smoothing. Time-of-flight and point-spread-function modeling were applied, in addition to attenuation correction using CT maps.

### Assessment of the [^18^F]FDG PET/CT imaging

Two experienced nuclear medicine physicians (SC and Y-iK) reviewed the [^18^F]FDG PET/CT images to identify the visual findings and measure the quantitative parameters. For the visual findings, the PET visual grade of the anterior mediastinal mass was defined as 0 (no uptake at whole mass), 1 (less than thoracic aorta uptake), 2 (equal to thoracic aorta uptake), 3 (from more than thoracic aorta uptake to equal to liver uptake), or 4 (more than liver uptake). A PET central metabolic defect was defined as no [^18^F]FDG uptake in more than 70–80% of the anterior mediastinal mass. The CT shape was classified as round when the ratio between the long-axis and short-axis dimensions was less than 1.5, and oval when the ratio was greater than 1.5.

For the quantitative parameters, a volume-of-interest (VOI) was delineated to cover the anterior mediastinal mass. The maximum standardized uptake value (SUVmax) of the PET was calculated as “tumour activity/(injection dose/lean body mass obtained from body weight and height)” using Mirada DBX software (version 1.2.0.59: Mirada Medical Ltd). In combination with the CT, we measured the long-axis diameter and HU of the anterior mediastinal mass at the same level while obtaining the SUVmax.

### Statistical analysis

Patient age and quantitative parameters on [^18^F]FDG PET/CT are expressed as median and range. The differences between groups were analyzed using Pearson’s chi-squared test or the Mann-Whitney U-test. Receiver operating characteristics (ROC) analysis was employed to compare the diagnostic capabilities of the [^18^F]FDG PET/CT quantitative parameters to diagnose thymic cysts. Optimal cut-offs were defined as the exploratory cut-offs with highest accuracy according to Youden’s index. Areas under the ROC curves (AUCs) and their 95% confidence intervals (CIs) were calculated and compared using DeLong’s method. Statistical analysis was performed using MedCalc version 22.017 (MedCalc Software Ltd., Ostend, Belgium). A *P*-value less than 0.05 was considered to be significant.

## Results

### Patients

Among the finally included 70 patients, 18 had a thymic cyst and 52 had low-risk thymoma. The median age was 58.0 years and 32 patients were male (45.7%). Fifteen (21.4%) patients were symptomatic. Common symptoms were cough (10.0%), followed by chest pain (5.7%), dyspnea (2.8%), ocular myasthenia gravis (1.4%), and diplopia (1.4%). Thirteen (18.6%) patients had a concomitant or previous cancer, with these including thyroid cancer (8.6%), lung cancer (2.8%), hepatocellular carcinoma (2.8%), breast cancer (1.4%), bladder cancer (1.4%), and malignant nerve sheath tumour (1.4%). Comparisons between thymic cyst and low-risk thymoma showed no significant differences in age, gender, symptoms, and concomitant or previous malignancy (Table 1). The characteristics and [^18^F]FDG PET/CT findings of the patients with a thymic cyst are summarized in Supplementary Table 1.

**Table 1:** Patient profiles

Characteristics	Total (*n* = 70)	Thymic cyst (*n* = 18)	Low-risk thymoma (*n* = 52)	*P*-value
Age (years, median with range)	58.0 (17.0–76.0)	60.5 (17.0–72.0)	56.0 (27.0–76.0)	0.199
Gender (male: female)	32:38	6:12	26:26	0.225
Symptom positivity	15 (21.4%)	5 (27.8%)	10 (19.2%)	0.450
Concomitant or previous malignancy	13 (18.6%)	5 (27.8%)	8 (15.4%)	0.247

### Visual findings of [^18^F]FDG PET/CT

Fourteen cases of thymic cysts (94.4%) were classified as grade 0, 1, or 2, whereas all low-grade thymomas were classified as grade 2 or higher. The majority of thymic cysts (*n* = 17, 94.4%) exhibited a central metabolic defect, while in contrast, only six cases (11.5%) of low-risk thymoma showed a central metabolic defect. Statistically significant differences in visual grade and central metabolic defect were observed between thymic cysts and low-risk thymomas, with the *P*-values for both being < 0.001. Using a cut-off of equal to or less than PET visual grade 2 to differentiate thymic cysts from low-risk thymomas, the diagnostic sensitivity, specificity, and AUC were 94.4%, 90.4%, and 0.924, respectively. Using the presence of a PET central metabolic defect for the differentiation, the sensitivity, specificity, and AUC were 94.4%, 88.5%, and 0.915, respectively. In both thymic cyst and low-risk thymoma groups, the CT shape was predominantly oval, accounting for 55.6% and 59.6% of cases, respectively, with there being no statistically significant difference (Table 2).

**Table 2:** Comparison of [^18^F]FDG PET/CT visual findings

Findings	Total (*n* = 70)	Thymic cyst (*n* = 18)	Low-risk thymoma (*n* = 52)	*P*-value
PET visual grade				
0	10	10	0	< 0.001
1	4	4	0	
2	8	3	5	
3	20	1	19	
4	28	0	28	
PET central metabolic defect				
Positive	23	17	6	< 0.001
Negative	47	1	46	
CT shape				
Round	29	8	21	0.765
Oval	41	10	31	

*P < 0.05

### Quantitative analysis of [^18^F]FDG PET/CT

**Fig. 2:**
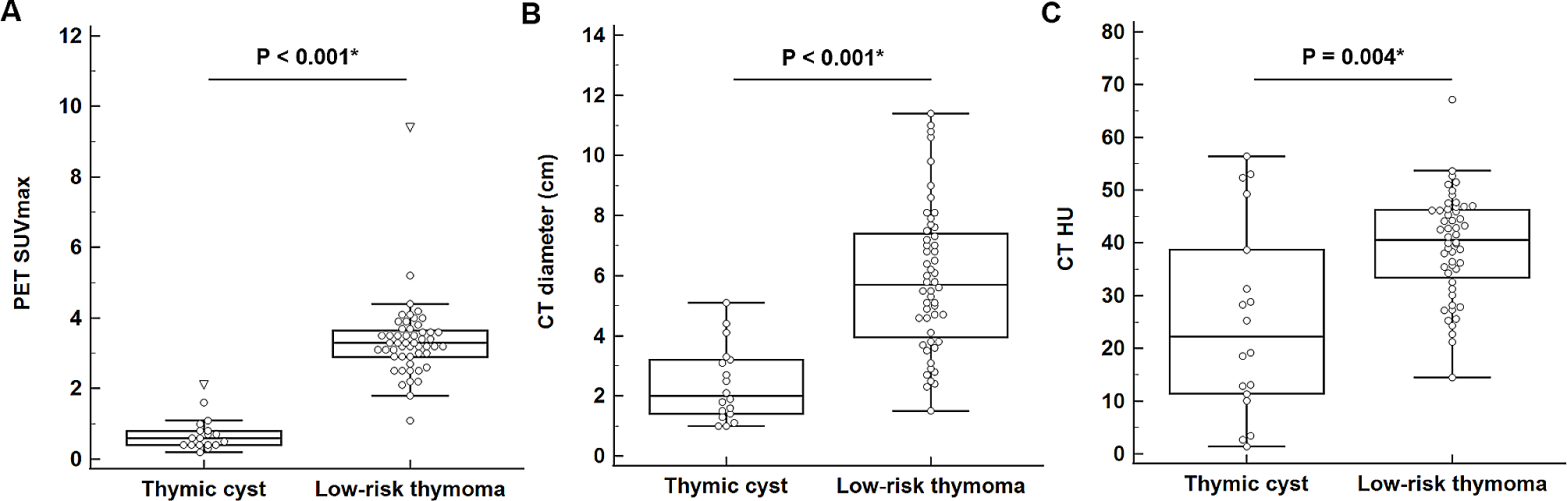
Comparison of [^18^F]FDG PET/CT quantitative parameters between thymic cyst and low-risk thymoma. **(A)** PET SUVmax (*P* < 0.001*), **(B)** CT diameter (*P* < 0.001*), and **(C)** CT HU (*P* = 0.004*) demonstrated significantly lower values in thymic cyst than in low-risk thymoma

**Fig. 3:**
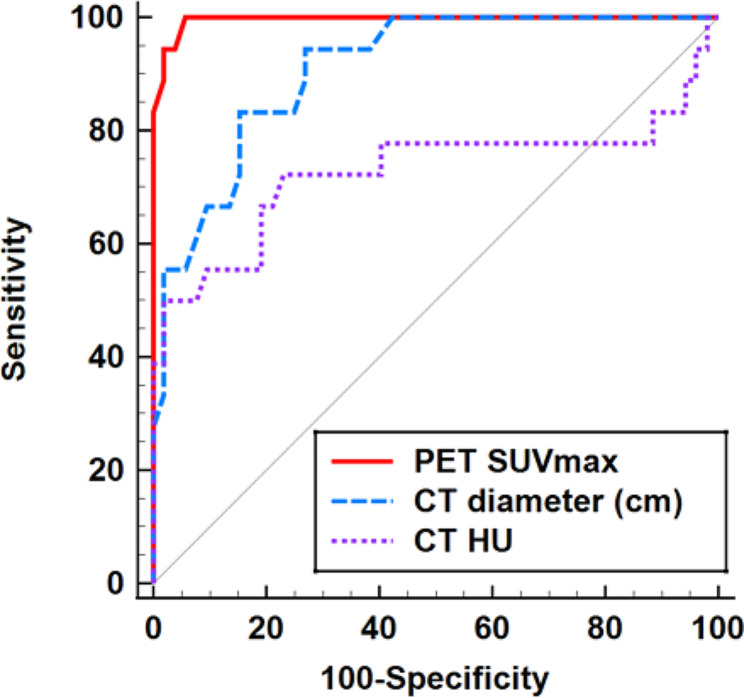
Receiver operating characteristics (ROC) curve analysis of diagnostic performance for thymic cyst with [^18^F]FDG PET/CT quantitative parameters. In the ROC curve analysis, PET SUVmax revealed the highest area under the curve (AUC) of 0.996 (*P* < 0.004). The sensitivity and specificity of PET SUVmax (cut-off of ≤ 2.1) were 100.0% and 94.2%, respectively

The quantitative [^18^F]FDG PET/CT parameters of PET SUVmax (*P* < 0.001), CT diameter (*P* < 0.001), and CT HU (*P* = 0.004) were significantly lower for thymic cysts than for low-risk thymomas (Fig. 2; Table 3). The ROC curve analysis revealed that PET SUVmax ≤ 2.1 yielded the highest AUC of 0.996 (95% CI: 0.940–1.000), achieving 100.0% sensitivity and 94.2% specificity in diagnosing thymic cysts (Fig. 3).

**Table 3:** Comparison of [^18^F]FDG PET/CT quantitative parameters

Parameters	Total (*n* = 70)	Thymic cyst (*n* = 18)	Low-risk thymoma (*n* = 52)	*P*-value
PET SUVmax	3.1 (0.2–9.4)	0.6 (0.2–2.1)	3.3 (1.1–9.4)	< 0.001*
CT diameter (cm)	4.8 (1.0–10.4)	2.0 (1.0–5.1)	5.7 (1.5–10.4)	< 0.001*
CT HU	0.6 (1.4–67.2)	22.3 (1.4–56.4)	40.6 (14.5–67.2)	0.004*

SUVmax = maximum standardized uptake value; HU = Hounsfield units

* *P* < 0.05

**Fig. 4:**
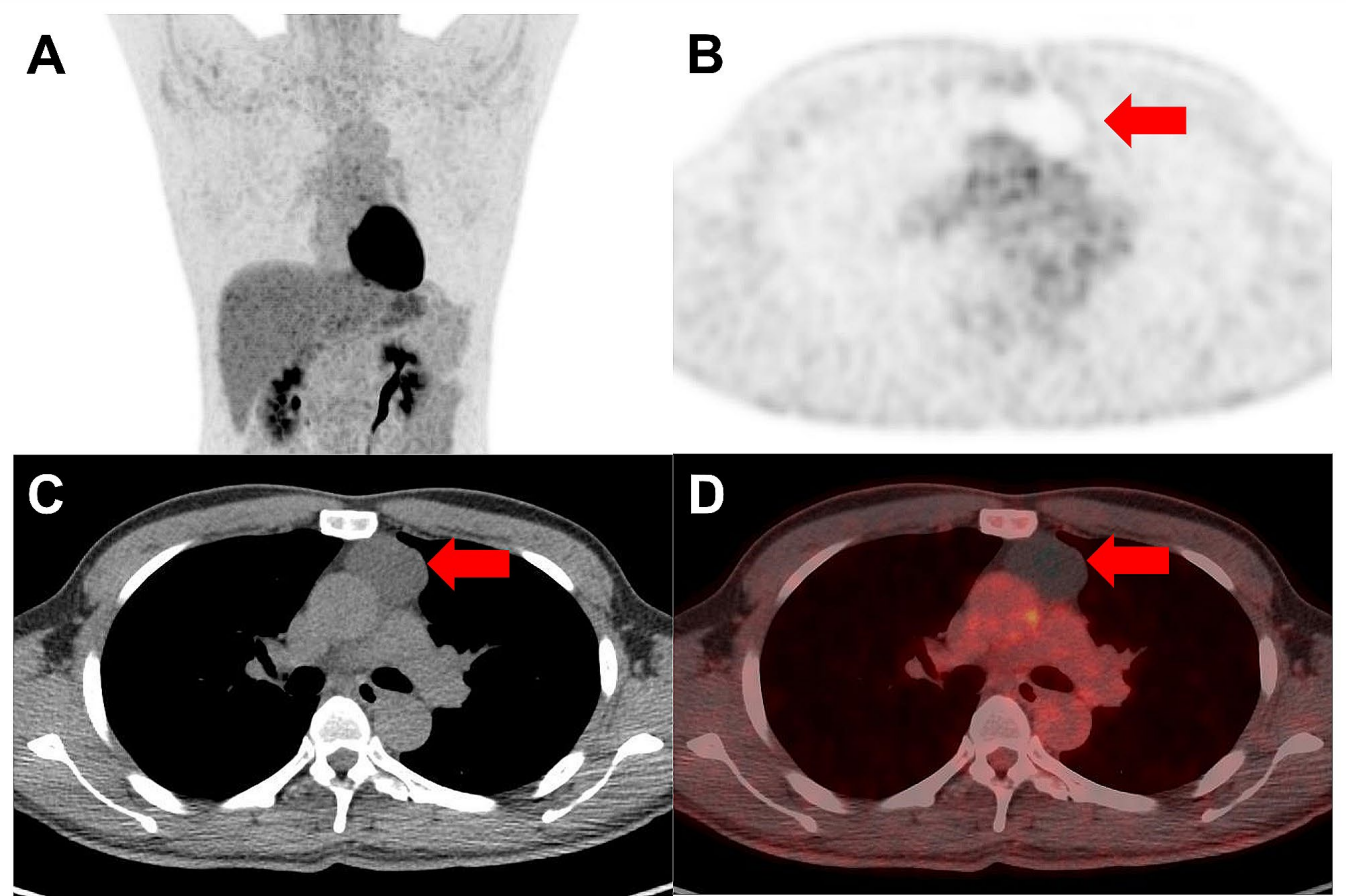
Representative [^18^F]FDG PET/CT from a 45-year-old man with a thymic cyst (red arrows). **(A)** Maximal intensity projection and **(B)** axial PET images show an anterior mediastinal mass with grade 1 uptake, central metabolic defect, and SUVmax of 1.0. **(C)** Axial CT demonstrates an anterior mediastinal mass with an oval shape, diameter of 4.4 cm, and attenuation of 12.5 HU. **(D)** Axial PET/CT fusion image

**Fig. 5:**
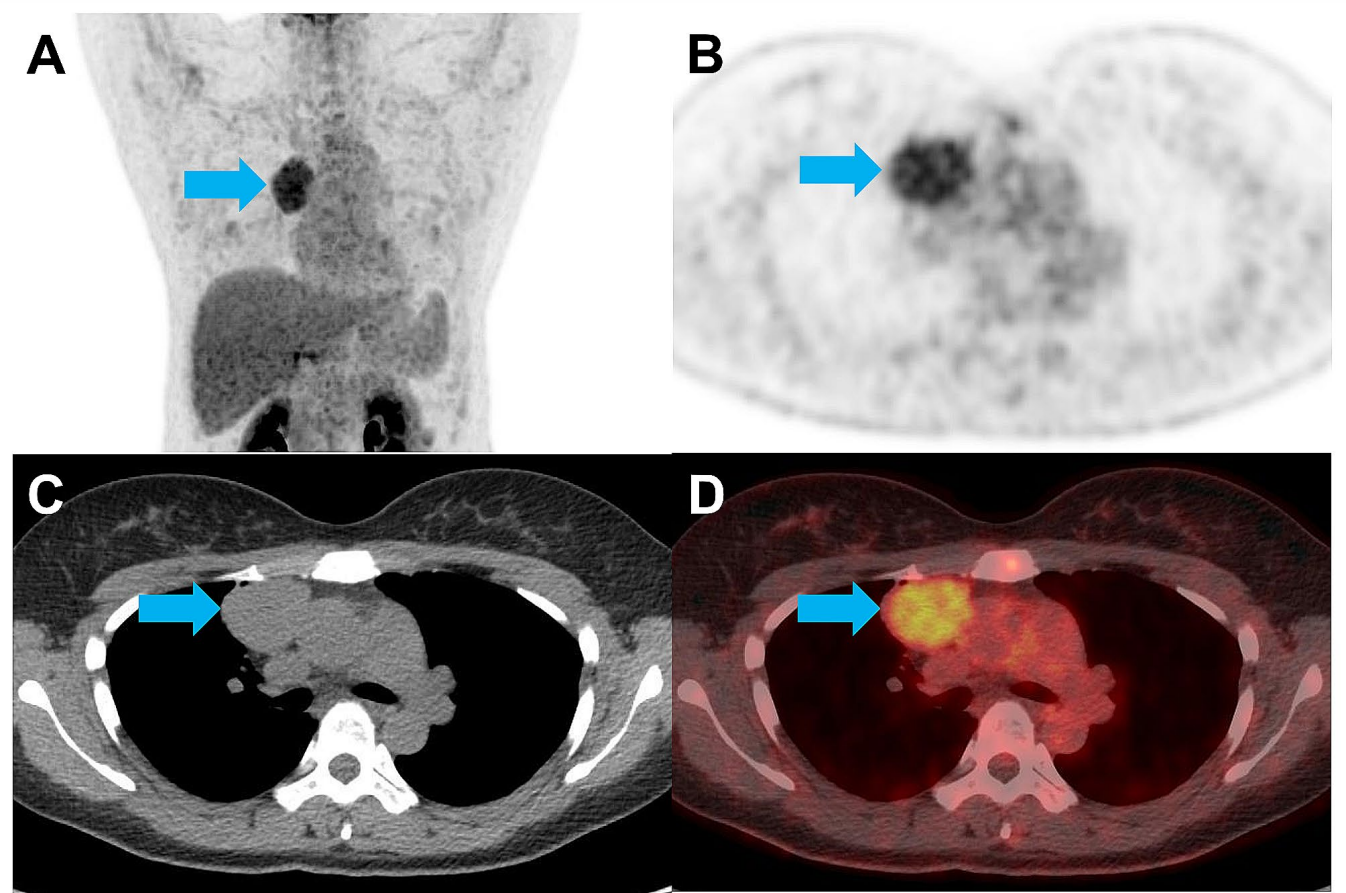
Representative [^18^F]FDG PET/CT from a 38-year-old woman with low-risk thymoma (blue arrows). **(A)** Maximal intensity projection and **(B)** axial PET images show an anterior mediastinal mass with grade 4 uptake, no central metabolic defect, and SUVmax of 3.9. **(C)** Axial CT demonstrates an anterior mediastinal mass with a round shape, diameter of 4.4 cm, and attenuation of 42.7 HU. **(D)** Axial PET/CT fusion image

Comparison of the AUCs revealed that SUVmax showed a significantly higher AUC than CT diameter (*P* = 0.009) and CT HU (*P* = 0.004) (Table 4). Representative cases of thymic cyst and low-risk thymoma are demonstrated in Figs. 4 and 5, respectively.

**Table 4:** ROC curve analysis results of [^18^F]FDG PET/CT quantitative parameters in the diagnosis of thymic cyst

Parameter	AUC (95% CI)	Cut-off	Sensitivity (%)	Specificity (%)	*p*-value	*P*-value (Comparison with AUC of SUVmax)
PET SUVmax	0.996 (0.940–1.000)	≤ 2.1	100.0	94.2	< 0.001*	(-)
CT diameter (cm)	0.910 (0.817–0.965)	≤ 3.3	83.3	84.6	< 0.001*	0.009*
CT HU	0.728 (0.608–0.827)	≤ 31.3	72.2	76.9	0.013*	0.004*

CI = confidence interval; ROC = receiver operating characteristics; AUC = area under the curve; SUVmax = maximum standardized uptake value; HU = Hounsfield units

*P < 0.05

## Discussion

This study investigated the diagnostic performance of visual findings and quantitative parameters of [^18^F]FDG PET/CT for distinguishing between thymic cysts and low-risk thymoma. PET visual grade and PET central metabolic defect were significant visual findings of [^18^F]FDG PET/CT, but CT shape was not. PET SUVmax, CT diameter, and CT attenuation in HU were significant quantitative parameters of [^18^F]FDG PET/CT, with PET SUVmax being the parameter with the highest AUC. Visual and quantitative PET findings of [^18^F]FDG PET/CT were shown to be useful for diagnosing thymic cysts. As far as we are aware, this study is the first to compare [^18^F]FDG PET/CT findings between thymic cyst and low-risk thymoma. By distinguishing between thymic cysts and low-risk thymomas, we anticipate reducing unnecessary invasive surgeries for patients with thymic cysts.

Previous studies on [^18^F]FDG PET/CT showed SUVmax to have better diagnostic utility than morphologic features from CT [20–22]. However, these previous studies included all types of TETs as a control (low-risk thymoma, high-risk thymoma, and thymic carcinomas), and demonstrated distinctive PET and CT features among the types [20, 23]. Despite its better diagnostic capability, additional work may be needed to facilitate measurement of SUVmax, whereas in contrast, the PET visual grade can be obtained more easily and conveniently than quantitative PET parameters. The PET visual grade system is also used to evaluate treatment effectiveness in a similar manner to Deauville’s score. The Deauville criteria, a reproducible five-point scale, are widely utilized for interpreting interim PET/CT scans in Hodgkin lymphoma and diffuse large B-cell lymphoma [24]. The two primary reference organs in this criteria system are the liver and the mediastinum. Following the model of these five-point criteria, we adopted a comparable methodology for classifying thymic masses, assessing their metabolic activity in relation to organs like the liver and mediastinum. The majority of thymic cysts in our study showed FDG uptake equal to or lower than that of the mediastinum, resulting in a statistically significant difference in comparison with low-risk thymoma, and a PET visual grade cut-off value of two was adopted.

Although thymic cysts typically show water-like attenuation (HU < 20), atypical protein-rich thymic cysts with solid density may be difficult to differentiate from thymomas featuring low vascularity. Hemorrhage or inflammation within the cyst may result in the formation of cyst fluid that is rich in proteins, which may show soft tissue density on chest imaging studies. In our data, the median and cut-off values for CT attenuation for thymic cysts were 22.3 and 31.3 HU, respectively; values that are higher than the attenuation of pure water (0 HU). In certain cases, discriminating cystic lesions from solid masses using only CT values proves to be challenging. Anterior mediastinal masses judged to require surgical resection may exhibit morphologic features on CT that are closer to those of thymoma than thymic cyst, with observation being the general clinical decision for thymic cyst. If anterior mediastinal masses cannot be distinguished from thymomas on the basis of their morphologic features on CT images and have attenuation higher than 20 HU, additional information may be needed to determine the need for surgery, rather than an invasive biopsy. Cyst components usually exhibit a metabolic defect on [^18^F]FDG PET/CT [25]. Our study revealed that a visual metabolic defect in an anterior mediastinal mass on PET may indicate a thymic cyst. However, when comparing the diagnostic efficacy of central metabolic defect with SUVmax and the visual grade of PET, the presence of a central metabolic defect appears to have limited value in diagnosing thymic cysts.

Recent studies reported that the median SUVmax of thymic cysts was lower than that of thymomas in surgically-resected thymic abnormalities, and that SUVmax demonstrated comparable diagnostic performance to the morphological features observed on CT [22, 23]. In our study, the median SUVmax was significantly lower in thymic cysts that in low-risk thymomas (0.6 vs. 3.3, *P* < 0.001). Comparisons of the ROC curves of our [^18^F]FDG PET/CT-based findings demonstrated that SUVmax was more effective for diagnosing thymic cysts than CT diameter and CT HU measurement. Upon reviewing discordant cases between PET SUVmax and CT diameter, we found that all four patients with PET SUVmax equal to or less than 2.1 (suggesting thymic cysts) and CT diameter greater than 3.3 cm (indicating low-risk thymomas) were diagnosed with thymic cysts. Additionally, among nine patients with PET SUVmax greater than 2.1 (suggesting low-risk thymomas) and CT diameter equal to or less than 3.3 cm (indicating thymic cysts), the final diagnoses were all low-risk thymomas. 

For small thymic cysts, observation without surgical resection is recommended unless there is evidence of growth or a very large cystic mass. Conversely, when a thymic lesion is definitively diagnosed as thymoma, early surgical resection or minimally invasive surgery are strongly advised. The discrimination between thymic cysts and thymoma is challenging in small thymic abnormalities. In previous studies, nontherapeutic thymectomy rates varied between 22% and 68% [17, 18], and thymic cysts were the most frequent diagnoses in nontherapeutic thymectomies [18]. Because of pseudo-enhancement caused by their proximity to the thoracic aorta and sternum, thymic cysts can be incorrectly diagnosed as small thymoma on CT imaging. Non-invasive thymoma (such as low-risk thymoma) and thymic cyst usually show a well-defined marginal contour and homogenous CT attenuation. In our study, both thymic cysts and low-risk thymomas exhibited a higher frequency of an oval shape on CT images, with no statistically significant difference between them.

This study has some limitations. As this study was a retrospective single center study, the risk of selection bias exists. Pathology results were unavailable for cases opting for close observation rather than surgery, and therefore only those cases that underwent resection were included in the analysis. In addition, the number of thymic cyst patients was small because thymic cysts are a rare disease and thymic lesions do not commonly undergo preoperative [^18^F]FDG PET/CT. It is necessary to perform further studies with larger populations and multicentre prospective designs to provide robust confirmation of our findings.

## Conclusions

In conclusion, on [^18^F]FDG PET/CT, decreased uptake equal to or less than mediastinum, the presence of a central metabolic defect, and low SUVmax (≤ 2.1) were findings more commonly observed in thymic cysts than in low-risk thymoma. [^18^F]FDG PET/CT could aid in diagnosing thymic cysts, potentially reducing the need for unnecessary invasive surgery.

### Electronic supplementary material

Below is the link to the electronic supplementary material.

Supplementary Material 1

## Data Availability

The datasets used and analyzed during the current study are available from the corresponding author upon reasonable request.

## Abbreviations

LDCT: low-dose chest computed tomography
TET: thymic epithelial tumour
WHO: World Health Organization
HU: Hounsfield units
FDG: fluorodeoxyglucose
PET: positron emission tomography
CT: computed tomography
VOI: volume-of-interest
SUVmax: maximum standardized uptake value
ROC: rceiver operating characteristics
AUC: Areas under the ROC curves
CI: confidence interval
