# Supplementary material for: Distinguishing thymic cysts from low-risk thymomas via [18F]FDG PET/CT
Source: EJNMMI Res. 2024 May 3;14:45. doi: 10.1186/s13550-024-01108-3 (PMC11068711; doi:10.1186/s13550-024-01108-3)
Supplement: Supplementary file 1 — Supplementary Material 1 [file 13550_2024_1108_MOESM1_ESM.docx]

**SUPPLEMENTARY TABLE**

**Supplementary Table 1.** Summary of [^18^F]FDG PET/CT findings of patients with a thymic cyst

| No. | Age | Gender | Symptom positivity | Concomitant  or previous malignancy | PET  visual  grade | PET central  metabolic  defect | CT  shape | PET  SUVmax | CT diameter (cm) | CT  HU |
| --- | --- | --- | --- | --- | --- | --- | --- | --- | --- | --- |
| 1 | 67 | Female | Positive | Yes | 0 | Positive | Round | 0.3 | 2.5 | -4.0 |
| 2 | 17 | Female | Negative | No | 3 | Positive | Oval | 0.9 | 4.1 | 30.3 |
| 3 | 66 | Female | Negative | Yes | 0 | Positive | Oval | 0.6 | 3.3 | 24.0 |
| 4 | 67 | Male | Negative | No | 0 | Positive | Oval | 0.2 | 3.1 | 49.0 |
| 5 | 65 | Male | Negative | Yes | 0 | Positive | Round | 0.7 | 1.0 | 13.0 |
| 6 | 59 | Female | Negative | No | 0 | Positive | Oval | 0.3 | 3.2 | 58.3 |
| 7 | 60 | Female | Positive | Yes | 0 | Positive | Round | 0.5 | 1.1 | 19.5 |
| 8 | 55 | Female | Positive | No | 0 | Positive | Oval | 0.2 | 2.7 | 8.5 |
| 9 | 54 | Male | Negative | No | 2 | Positive | Round | 0.6 | 1.3 | 54.9 |
| 10 | 58 | Female | Negative | No | 2 | Positive | Round | 0.6 | 2.1 | 11.7 |
| 11 | 60 | Female | Positive | No | 1 | Positive | Oval | 0.2 | 1.9 | 45.6 |
| 12 | 61 | Female | Negative | No | 0 | Positive | Round | 0.2 | 1.6 | 24.2 |
| 13 | 72 | Female | Negative | No | 0 | Positive | Oval | 0.4 | 1.4 | 14.3 |
| 14 | 64 | Male | Negative | No | 0 | Positive | Oval | 0.3 | 1.8 | 55.8 |
| 15 | 45 | Male | Negative | No | 1 | Positive | Oval | 0.2 | 4.4 | 12.5 |
| 16 | 55 | Female | Negative | No | 2 | Positive | Oval | 0.2 | 5.1 | 9.4 |
| 17 | 64 | Female | Negative | No | 1 | Negative | Round | 0.7 | 1.0 | 48.3 |
| 18 | 72 | Male | Positive | Yes | 1 | Positive | Round | 0.4 | 1.5 | 37.3 |

SUVmax = maximum standardized uptake value; HU = Hounsfield units
